# Supplementary material for: Association between maternal high-risk fertility behaviour and perinatal mortality in Bangladesh: Evidence from the Demographic and Health Survey
Source: PLoS One. 2023 Nov 27;18(11):e0294464. doi: 10.1371/journal.pone.0294464 (PMC10681254; doi:10.1371/journal.pone.0294464)
Supplement: S1 Table — (DOCX) [file pone.0294464.s001.docx]

Supplementary Table 1: Association between perinatal mortality and high risk fertility behaviour adjusted for individual, household and community level factors

|  | Individual level, OR (95% CI) | Individual and household level, OR (95% CI) | Individual, household, and community level, OR (95% CI) |
| --- | --- | --- | --- |
| High risk fertility behaviour |  |  |  |
| No (ref) | 1.00 | 1.00 | 1.00 |
| Yes | 6.23 (4.80-8.11)^**^ | 3.52 (1.99-4.53)^**^ | 1.87 (1.61-2.14)^**^ |
| Respondents’ education |  |  |  |
| No education | 1.00 | 1.00 | 1.00 |
| Primary education | 1.93 (1.24-3.03)^**^ | 1.80 (1.15-2.82)^**^ | 1.82 (1.16-2.85) ^**^ |
| Secondary education | 1.86 (1.19-2.90) ^**^ | 1.77 (1.13-2.78)^**^ | 1.78 (1.13-2.80) ^**^ |
| Higher education | 2.48 (1.50-4.11)^**^ | 2.65 (1.54-4.56)^**^ | 2.66 (1.53-4.60)^**^ |
| Respondents’ working status |  |  |  |
| No | 1.00 | 1.00 | 1.00 |
| Yes | 1.14 (0.93-1.40) | 1.19 (0.96-1.47) | 1.17 (0.94-1.45) |
| Respondents’ partner occupation |  |  |  |
| Agricultural worker |  | 1.00 | 1.00 |
| Bule color worker |  | 1.29 (0.99-1.70) | 1.27 (0.96-1.66) |
| White color worker |  | 0.80 (0.44-1.47) | 0.80 (0.43-1.45) |
| Pink color worker |  | 0.99 (0.70-1.39) | 0.98 (0.69-1.38) |
| Others |  | 1.22 (0.42-3.54) | 1.23 (0.42-3.56) |
| Wealth quintile |  |  |  |
| Poorest |  | 1.00 | 1.00 |
| Poorer |  | 1.17 (0.85-1.59) | 1.17 (0.94-1.46) |
| Middle |  | 1.24 (0.90-1.72) | 1.21 (0.87-1.68) |
| Richer |  | 1.09 (0.78-1.53) | 1.05 (0.74-1.49) |
| Richest |  | 1.04 (0.70-1.53) | 0.956 (0.63-1.47) |
| Place of residence |  |  |  |
| Urban |  |  | 1.00 |
| Rural |  |  | 0.92 (0.72-1.19) |
| Region of residence |  |  |  |
| Barishal |  |  | 1.00 |
| Chattogram |  |  | 1.18 (0.77-1.81) |
| Dhaka |  |  | 1.18 (0.75-1.84) |
| Khulna |  |  | 0.99 (0.61-1.62) |
| Mymensingh |  |  | 1.19 (0.76-1.86) |
| Rajshahi |  |  | 1.22 (0.77-1.92) |
| Rangpur |  |  | 0.98 (0.61-1.56) |
| Sylhet |  |  | 0.86 (0.55-1.36) |

Note: ^**^p<0.01, ^*^p<0.05
