# Supplementary material for: Association between maternal high-risk fertility behaviour and perinatal mortality in Bangladesh: Evidence from the Demographic and Health Survey
Source: PLoS One. 2023 Nov 27;18(11):e0294464. doi: 10.1371/journal.pone.0294464 (PMC10681254; doi:10.1371/journal.pone.0294464)
Supplement: S2 Table — (DOCX) [file pone.0294464.s002.docx]

Supplementary Table 2: Association between perinatal mortality and number of high-risk fertility behaviour adjusted for individual, household and community level factors

|  | Individual level, OR (95% CI) | Individual and household level, OR (95% CI) | Individual, household, and community level, OR (95% CI) |
| --- | --- | --- | --- |
| High risk fertility behaviour |  |  |  |
| No (ref) | 1.00 | 1.00 | 1.00 |
| Single | 5.70 (4.36-7.47)^**^ | 2.76 (1.88-3.45)^**^ | 1.77 (1.50-2.05) |
| Multiple | 9.04 (6.47-12.65)^**^ | 4.56 (3.56-5.65)^**^ | 2.30 (1.96-2.64) |
| Respondents’ education |  |  |  |
| No education | 1.00 | 1.00 | 1.00 |
| Primary education | 2.09 (1.33-3.26)^**^ | 1.96 (1.25-3.09)^**^ | 1.99 (1.26-3.13)^**^ |
| Secondary education | 2.05 (1.31-3.22)^**^ | 2.00 (1.26-3.15)^**^ | 2.07 (1.27-3.18)^**^ |
| Higher education | 2.67 (1.61-4.44)^**^ | 2.94 (1.70-5.08)^**^ | 2.94 (1.69-5.11)^**^ |
| Respondents’ working status |  |  |  |
| No | 1.00 | 1.00 | 1.00 |
| Yes | 1.14 (0.93-1.40) | 1.18 (0.95-1.46) | 1.15 (0.93-1.43) |
| Respondents’ partner occupation |  |  |  |
| Agricultural worker |  | 1.00 | 1.00 |
| Bule color worker |  | 1.32 (1.01-1.74)^*^ | 1.29 (0.98-1.70) |
| White color worker |  | 0.80 (0.44-1.46) | 0.79 (0.43-1.44) |
| Pink color worker |  | 0.98 (0.70-1.40) | 0.97 (0.68-1.37) |
| Others |  | 1.13 (0.39-3.29) | 1.13 (0.38-3.28) |
| Wealth quintile |  |  |  |
| Poorest |  | 1.00 | 1.00 |
| Poorer |  | 1.15 (0.85-1.57) | 1.16 (0.85-1.57) |
| Middle |  | 1.22 (0.88-1.69) | 1.18 (0.85-1.64) |
| Richer |  | 1.07 (0.76-1.49) | 1.02 (0.72-1.44) |
| Richest |  | 1.01 (0.69-1.50) | 0.94 (0.62-1.44) |
| Place of residence |  |  |  |
| Urban |  |  | 1.00 |
| Rural |  |  | 0.92 (0.72-1.18) |
| Region of residence |  |  |  |
| Barishal |  |  | 1.00 |
| Chattogram |  |  | 1.19 (0.78-1.82) |
| Dhaka |  |  | 1.21 (0.77-1.88) |
| Khulna |  |  | 1.02 (0.63-1.67) |
| Mymensingh |  |  | 1.23 (0.79-1.92) |
| Rajshahi |  |  | 1.25 (0.80-1.98) |
| Rangpur |  |  | 1.01 (0.63-1.61) |
| Sylhet |  |  | 0.85 (0.54-1.35) |

Note: ^**^p<0.01, ^*^p<0.05
