# Supplementary material for: Association between maternal high-risk fertility behaviour and perinatal mortality in Bangladesh: Evidence from the Demographic and Health Survey
Source: PLoS One. 2023 Nov 27;18(11):e0294464. doi: 10.1371/journal.pone.0294464 (PMC10681254; doi:10.1371/journal.pone.0294464)
Supplement: S3 Table — (DOCX) [file pone.0294464.s003.docx]

Supplementary Table 3: Association between perinatal mortality and no high risk fertility behaviour adjusted for individual, household and community level factors

|  | Individual level, OR (95% CI) | Individual and household level, OR (95% CI) | Individual, household, and community level, OR (95% CI) |
| --- | --- | --- | --- |
| High risk fertility behaviour |  |  |  |
| No (ref) | 1.00 | 1.00 | 1.00 |
| Yes | 0.38 (0.29-0.51)^**^ | 0.36 (0.27-0.49)^**^ | 0.36 (0.27-0.49)^**^ |
| Respondents’ education |  |  |  |
| No education | 1.00 | 1.00 | 1.00 |
| Primary education | 1.73 (1.11-2.71)^**^ | 1.63 (1.04-2.55) | 1.65 (1.05-2.58)^**^ |
| Secondary education | 1.40 (0.90-2.17)^**^ | 1.36 (0.87-2.13) | 1.37 (0.87-2.15) |
| Higher education | 1.16 (0.71-1.90)^**^ | 1.26 (0.74-2.14) | 1.28 (0.75-2.18) |
| Respondents’ working status |  |  |  |
| No | 1.00 | 1.00 | 1.00 |
| Yes | 1.29 (1.05-1.57) | 1.32 (1.07-1.63)^**^ | 1.31 (1.05-1.62)^**^ |
| Respondents’ partner occupation |  |  |  |
| Agricultural worker |  | 1.00 | 1.00 |
| Bule color worker |  | 1.16 (0.80-1.48) | 1.14 (0.86-1.49) |
| White color worker |  | 1.13 (0.82-1.56) | 0.78 (0.43-1.42) |
| Pink color worker |  | 1.02 (0.73-1.42) | 0.96 (0.68-1.35) |
| Others |  | 0.94 (0.64-1.37) | 1.17 (0.41-3.34) |
| Wealth quintile |  |  |  |
| Poorest |  | 1.00 | 1.00 |
| Poorer |  | 1.09 (0.80-1.48) | 1.09 (0.80-1.48) |
| Middle |  | 1.13 (0.81-1.55) | 1.10 (0.80-1.53) |
| Richer |  | 1.02 (0.73-1.42) | 0.98 (0.69-1.38) |
| Richest |  | 0.94 (0.64-1.37) | 0.88 (0.58-1.33) |
| Place of residence |  |  |  |
| Urban |  |  | 1.00 |
| Rural |  |  | 0.93 (0.73-1.19) |
| Region of residence |  |  |  |
| Barishal |  |  | 1.00 |
| Chattogram |  |  | 1.24 (0.82-1.88) |
| Dhaka |  |  | 1.13 (0.73-1.76) |
| Khulna |  |  | 0.94 (0.58-1.52) |
| Mymensingh |  |  | 1.13 (0.73-1.75) |
| Rajshahi |  |  | 1.22 (0.78-1.91) |
| Rangpur |  |  | 0.98 (0.62-1.56) |
| Sylhet |  |  | 0.87 (0.56-1.37) |

Note: ^**^p<0.01, ^*^p<0.05
