# Supplementary material for: Association between maternal high-risk fertility behaviour and perinatal mortality in Bangladesh: Evidence from the Demographic and Health Survey
Source: PLoS One. 2023 Nov 27;18(11):e0294464. doi: 10.1371/journal.pone.0294464 (PMC10681254; doi:10.1371/journal.pone.0294464)
Supplement: Attachment — Submitted filename: PONE-D-23-23194_TM.pdf [file pone.0294464.s004.pdf]

PLOS ONE

Association between maternal high-risk fertility behaviour and perinatal mortality in Bangladesh: evidence from the Demographic and Health Survey

--Manuscript Draft--

**Manuscript Number:**	PONE-D-23-23194
**Article Type:**	Research Article
**Full Title:**	Association between maternal high-risk fertility behaviour and perinatal mortality in Bangladesh: evidence from the Demographic and Health Survey
**Short Title:**	High risk fertility behaviour and perinatal mortality in Bangladesh
**Corresponding Author:**	Md. Nuruzzaman Khan<br>Jatiya Kabi Kazi Nazrul Islam University<br>Mymensingh, Bangladesh, BANGLADESH
**Keywords:**	High-risk fertility behaviour; adverse pregnancy characteristics; perinatal mortality; contraception; Bangladesh
**Abstract:**	Background<br><br>High risk fertility behaviours including pregnancy early or late in the reproductive life course, higher parity and short birth intervals are ongoing concerns in Low- and Middle-Income Countries (LMICs) such as Bangladesh. Although such factors have been identified as major risk factors for perinatal mortality, there has been a lack of progress in the area despite the implementation of the Millennium and Sustainable Development Goals. We therefore explored the effects of high risk maternal fertility behaviour on the occurrence of perinatal mortality in Bangladesh.<br><br>Methods<br><br>A total of 8,930 singleton pregnancies of seven or more months gestation were extracted from 2017/18 Bangladesh Demographic and Health Survey for analysis. Perinatal mortality was the outcome variable (yes, no) and the primary exposure variable was high-risk fertility behaviour in the previous five years (yes, no). The association between the exposure and outcome variable was determined using a mixed-effect multilevel logistic regression model, adjusted for covariates.<br><br>Results<br><br>Forty-six percent of the total births that occurred in the five years preceding the survey were high-risk. After adjusting for potential confounders, a 1.87 times (aOR, 1.87, 95% CI, 1.61-2.14) higher odds of perinatal mortality was found among women with any high-risk fertility behaviour as compared to women having no high-risk fertility behaviours. The odds of perinatal mortality were also found to increase in line with an increasing number of high-risk behaviour. A 1.77 times (95% CI, 1.50-2.05) increase in odds of perinatal mortality was found among women with single high-risk fertility behaviour and a 2.30 times (95% CI, 1.96-2.64) increase in odds was found among women with multiple high-risk fertility behaviours compared to women with no high-risk fertility behaviour.<br><br>Conclusion<br><br>Women's high-risk fertility behaviour is an important predictor of perinatal mortality in Bangladesh. Increased contraceptive use to allow appropriate birth spacing, educational interventions around the potential risks associated with high risk fertility behaviour (including short birth interval) in future pregnacies, and improved continuity of maternal healthcare service use among this population are required to improve birth outcomes in Bangladesh.
**Order of Authors:**	Md. Nuruzzaman Khan
	Melissa L. Harris, Ph.D.

Powered by Editorial Manager® and ProduXion Manager® from Aries Systems Corporation

Additional Information:	
**Question**	**Response**
**Financial Disclosure**<br><br>Enter a financial disclosure statement that describes the sources of funding for the work included in this submission. Review the submission guidelines for detailed requirements. View published research articles from *PLOS ONE* for specific examples.<br><br>This statement is required for submission and **will appear in the published article** if the submission is accepted. Please make sure it is accurate.<br><br>**Unfunded studies**<br>Enter: *The author(s) received no specific funding for this work.*<br><br>**Funded studies**<br>Enter a statement with the following details:<br>• Initials of the authors who received each award<br>• Grant numbers awarded to each author<br>• The full name of each funder<br>• URL of each funder website<br>• Did the sponsors or funders play any role in the study design, data collection and analysis, decision to publish, or preparation of the manuscript?<br>• **NO** - Include this sentence at the end of your statement: *The funders had no role in study design, data collection and analysis, decision to publish, or preparation of the manuscript.*<br>• **YES** - Specify the role(s) played.<br><br>* typeset	The author(s) received no specific funding for this work.
**Competing Interests**<br><br>Use the instructions below to enter a competing interest statement for this submission. On behalf of all authors, disclose any competing interests that could be perceived to bias this work—acknowledging all financial support and any other relevant financial or non-financial competing interests.	The authors have declared that no competing interests exist.

Powered by Editorial Manager® and ProduXion Manager® from Aries Systems Corporation

This statement is required for submission and **will appear in the published article** if the submission is accepted. Please make sure it is accurate and that any funding sources listed in your Funding Information later in the submission form are also declared in your Financial Disclosure statement.<br><br>View published research articles from *PLOS ONE* for specific examples.<br><br>**NO authors have competing interests**<br><br>Enter: *The authors have declared that no competing interests exist.*<br><br>**Authors with competing interests**<br><br>Enter competing interest details beginning with this statement:<br><br>*I have read the journal's policy and the authors of this manuscript have the following competing interests: [insert competing interests here]*<br><br>* typeset	
**Ethics Statement**<br><br>Enter an ethics statement for this submission. This statement is required if the study involved:<br><br>• Human participants<br>• Human specimens or tissue<br>• Vertebrate animals or cephalopods<br>• Vertebrate embryos or tissues<br>• Field research<br><br>Write "N/A" if the submission does not require an ethics statement.<br><br>General guidance is provided below. Consult the submission guidelines for detailed instructions. **Make sure that all information entered here is included in the**	The survey analysed was approved by the institutional review board of ICF and the National Research Ethics Committee of the Bangladesh Medical Research Council. Informed consent was obtained from all participants. All necessary patient/participant consent has been obtained and the appropriate institutional forms have been archived. No separate ethical approval was required to conduct this study. We obtained permission to access this survey and conduct this research. All methods were performed in accordance with the relevant guidelines and regulations.

**Methods section of the manuscript.**<br><br>**Format for specific study types**<br><br>**Human Subject Research (involving human participants and/or tissue)**<br>• Give the name of the institutional review board or ethics committee that approved the study<br>• Include the approval number and/or a statement indicating approval of this research<br>• Indicate the form of consent obtained (written/oral) or the reason that consent was not obtained (e.g. the data were analyzed anonymously)<br><br>**Animal Research (involving vertebrate animals, embryos or tissues)**<br>• Provide the name of the Institutional Animal Care and Use Committee (IACUC) or other relevant ethics board that reviewed the study protocol, and indicate whether they approved this research or granted a formal waiver of ethical approval<br>• Include an approval number if one was obtained<br>• If the study involved *non-human primates*, add *additional details* about animal welfare and steps taken to ameliorate suffering<br>• If anesthesia, euthanasia, or any kind of animal sacrifice is part of the study, include briefly which substances and/or methods were applied<br><br>**Field Research**<br><br>Include the following details if this study involves the collection of plant, animal, or other materials from a natural setting:<br>• Field permit number<br>• Name of the institution or relevant body that granted permission	
**Data Availability**<br><br>Authors are required to make all data underlying the findings described fully available, without restriction, and from the time of publication. PLOS allows rare exceptions to address legal and ethical concerns. See the PLOS Data Policy and FAQ for detailed information.	No - some restrictions will apply

A Data Availability Statement describing where the data can be found is required at submission. Your answers to this question constitute the Data Availability Statement and **will be published in the article**, if accepted.<br><br>**Important:** Stating 'data available on request from the author' is not sufficient. If your data are only available upon request, select 'No' for the first question and explain your exceptional situation in the text box.<br><br>Do the authors confirm that all data underlying the findings described in their manuscript are fully available without restriction?	
**Describe where the data may be found in full sentences. If you are copying our sample text, replace any instances of XXX with the appropriate details.**<br><br>• If the data are **held or will be held in a public repository**, include URLs, accession numbers or DOIs. If this information will only be available after acceptance, indicate this by ticking the box below. For example: *All XXX files are available from the XXX database (accession number(s) XXX, XXX.*).<br>• If the data are all contained **within the manuscript and/or Supporting Information files**, enter the following: *All relevant data are within the manuscript and its Supporting Information files.*<br>• If neither of these applies but you are able to provide **details of access elsewhere**, with or without limitations, please do so. For example:<br><br>*Data cannot be shared publicly because of [XXX]. Data are available from the XXX Institutional Data Access / Ethics Committee (contact via XXX) for researchers who meet the criteria for access to confidential data.*<br><br>*The data underlying the results*	The datasets used and analyzed in this study are available from the Measure DHS website: https://dhsprogram.com/data/available-datasets.cfm

Powered by Editorial Manager® and ProduXion Manager® from Aries Systems Corporation

presented in the study are available from (include the name of the third party and contact information or URL).<br>• This text is appropriate if the data are owned by a third party and authors do not have permission to share the data.<br><br>* typeset	
Additional data availability information:	

Powered by Editorial Manager® and ProduXion Manager® from Aries Systems Corporation

Click here to access/download;Manuscript;Manuscript.docx

Association between maternal high-risk fertility behaviour and perinatal mortality in Bangladesh: evidence from the Demographic and Health Survey

Md Nuruzzaman Khan[1,2],Ph.D, Melissa L. Harris[2], Ph.D.

[1]Deartment of Population Science, Jatiya Kabi Nazrul Islam University, Mymensingh, Bangladesh.

[2] Centre for Women's Health Research, University of Newcastle, Australia.

Short running title: High risk fertility behaviour and perinatal mortality in Bangladesh

Corresponding author

Md Nuruzzaman Khan, Ph.D.

Assistant Professor

Department of Population Science

Jatiya Kabi Kazi Nazrul Islam University, Trishal, Mymensingh, Bangladesh.

Email: sumonrupop@gmail.com

mdnuruzzaman.khan@uon.edu.au

Association between maternal high-risk fertility behaviour and perinatal mortality in Bangladesh: evidence from the Demographic and Health Survey

Abstract

Background

High risk fertility behaviours including pregnancy early or late in the reproductive life course, higher parity and short birth intervals are ongoing concerns in Low- and Middle-Income Countries (LMICs) such as Bangladesh. Although such factors have been identified as major risk factors for perinatal mortality, there has been a lack of progress in the area despite the implementation of the Millennium and Sustatinable Development Goals. We therefore explored the effects of high risk maternal fertility behaviour on the occurrence of perinatal mortality in Bangladesh.

Methods

A total of 8,930 singleton pregnancies of seven or more months gestation were extracted from 2017/18 Bangladesh Demographic and Health Survey for analysis. Perinatal mortality was the outcome variable (yes, no) and the primary exposure variable was high-risk fertility behaviour in the previous five years (yes, no). The association between the exposure and outcome variable was determined using a mixed-effect multilevel logistic regression model, adjusted for covariates.

Results

Forty-six percent of the total births that occurred in the five years preceding the survey were high-risk. After adjusting for potential confounders, a 1.87 times (aOR, 1.87, 95% CI, 1.61-2.14) higher odds of perinatal mortality was found among women with any high-risk fertility behaviour as compared to women having no high-risk fertility behaviours. The odds of perinatal mortality were also found to increase in line with an increasing number of high-risk behaviour. A 1.77 times (95% CI, 1.50-2.05) increase in odds of perinatal mortality was found among women with single high-risk fertility behaviour and a 2.30 times (95% CI, 1.96-2.64) increase in odds was found among

women with multiple high-risk fertility behaviours compared to women with no high-risk fertility behaviour.

Conclusion

Women's high-risk fertility behaviour is an important predictor of perinatal mortality in Bangladesh. Increased contraceptive use to allow appropriate birth spacing, educational interventions around the potential risks associated with high risk fertility behaviour (including short birth interval) in future pregnacies, and improved continuity of maternal healthcare service use among this population are required to improve birth outcomes in Bangladesh.

Keywords: High-risk fertility behaviour, adverse pregnancy characteristics, perinatal mortality, contraception, Bangladesh.

Introduction

An estimated 4.5 million perinatal mortality (stillbirths and early neonatal mortality) occur worldwide every year [1,2]. Perinatal mortality has been found to be particularly high in Low- and Middle-Income Countries (LMICs) and accounts for a large proportion of total child deaths. Among LMICs, the majority of deaths occurs in Sub-Saharan Africa (43 per 1000 live births) and South Asia (41 per 1000 live births) [3,4]. Particularly in Bangladesh perinatal mortaility accounts for 60% of the total under-five mortality [4,5]. Despite this, almost all perinatal mortaility are preventable [6,7]. Avoidable child mortality and its interventions (e.g., improving skilled delivery and neonatal care) are reflected in the Sustainable Development Goals (SDGs), which calls for ending preventable deaths of newborn babies (12 deaths per 1000 live births for neonatal mortality) and children younger than 5 years (25 deaths for 1000 live births for under-five mortality) by 2030 [3]. However, it is unlikely that LMICs such as Bangladesh will achieve these targets unless aggressive country-specific interventions are implemented [6].

Over the past 20 years, considerable improvements have occurred in developing healthcare facilities and accessing maternal healthcare services in LMICs such as Bangladesh, particularly as a result of the Millennium Development Goals period between 2000 to 2015 [8,9]. As such, the under-five mortality rate has declined significantly. However, this has not occurred for perinatal mortality [1,2]. For instance, the reduction of perinatal mortality (64 to 41 per 1000 live births) in Bangladesh was much slower than the reduction of under-five mortality (83 to 41 per 1000 live births) between 2000 and 2014 [4,10]. Women in Bangladesh are mostly not aware of the obstetric complications that prevent timely access to healthcare services and present to hospital with serious obstetric complications when successful intervention is reduced [11]. Availability of critical care for newborns is also limited in Bangladeshi healthcare facilities [12] and where services are available, distance represents a key barrier to access [13]. Consequently, deaths among new borns occur at substantially higher rates in Bangladesh compared to older aged children.

High-risk fertility behaviours, defined as pregnancy occurring early (i.e. <18 years) or later (>34 years) in the reproductive life course, pregnancies of a shorter interval (<24 months) and higher parity (i.e., more than two children) are common in LMICs [14]. In Bangladesh, the prevalence of early pregnancy (43%), short pregnancy interval (26%), higher parity (24%) are very high in Bangladesh and a significant proportion of pregnancies occur among women with more than one of these specific high risk behaviours [15-17]. These independent higher-risk fertility behaviour components have been found to interact with each other and adversely affect several maternal health outcomes, including chronic undernutrition and pregnancy complications, which is a risk factor for increased obstetric and newborn complications [14,18-21]. This along with healthcare facility level challenges may increase the occurrence of perinatal mortaility [14,18-21].

While it is posited that an association between higher risk fertility behaviour and perinatal mortality in LMICs such as Bangladesh exists, so far this understanding is limited. Available studies have mainly reported perinatal mortality outcomes in relation to disadvantaged socio-demographic characteristics (illiteracy, lower-income, and rurality) [4,22-24]. Only a few available studies in LMICs have determined the effects of particular high-risk fertility behaviours on perinatal mortality, such as, pregnancy before age 18 and short pregnancy interval [4,22]. A comprehensive understanding of the dynamic nature of perinatal mortality among women with high-risk fertility behaviour in general, and the compounding impact of multiple high-risk fertility behaviours, in particular, are still lacking in LMICs and to date no relevant study has been conducted in Bangaldesh. We, therefore, explored the effect of the high-risk fertility behaviour of mothers on perinatal mortality in Bangladesh adjusting for possible confounders.

Methods

Study design and sample

We analysed 2017/18 Bangladesh Demographic and Health Survey (BDHS) data. The BDHS is a nationally representative survey conducted every three years as part of the Demographic and Health Survey Program. The National Institute of Population Research and Training conducted this survey as a local body and representative of the Ministry of Health and Family Welfare of Bangladesh along with a local organization, the Mitra and Associate. Nationally representative households were selected to participate through a two-stage stratified random sampling process. A total of 675 primary sampling units (PSUs) were selected at the first stage of sampling covering each division and urban and rural area separately. The clusters were selected from a list of 293,579 PSUs generated by the Bangladesh Bureau of Statistics as part of the 2011 Bangladesh National Population Census. Three PSUs were excluded due to extreme floods and the survey was conducted in the remaining 672 PSUs. In the second stage of sampling, a fixed number of 30 households were randomly sampled from each of the selected PSUs. Of the 20,160 households selected, the survey was conducted by 19,457 households (96% participation rate). The overall criteria to be included in this survey were: (i) reproductive-aged (15-49 years) married women and (ii) resided permanently in the selected households or were a non-permanent resident of the selected households but resided there on the night before the survey. These criteria were met by 20,376 women. Of these, data were collected from 20,127 women resulting in a response rate of 98.8%. Women were asked to report on their reproductive history, contraception use, conception, pregnancy termination, stillbirths, and live births in the last five years. Women who reported at least one child within five years of the survey date, data on their children aged under five years were also collected. Informed written consent was obtained from all participants. Details of the sampling procedure have been published elsewhere [25].

Analytical sample

To examine perinatal mortality, this study extracted data from 8,959 pregnancies of 7 or more months gestation (>28 weeks of gestation). Of these, 29 twin births were excluded because of their different mortality risks for both children and mothers [25]. The final analysis sample consisted of 8,930 singleton births.

Outcome variable

The outcome variable for this study was perinatal mortality (yes, no) defined as the sum of stillbirths (fetal deaths in pregnancies with 7+ [>28 weeks] months of gestation) and early neonatal mortality (deaths within the first week of life). The relevant rate is calculated according to WHO guidelines.

Perinatal mortality=

$$\frac{\text{Pregnancy loss with 7+months of gestation (stillbirths)} + \text{Deaths within 7 days of live births (early neonatal deaths)}}{\text{Pregnancy loss with 7+months of gestation (stillbirths)} + \text{Number of live births}} \times 1{,}000$$

Exposure variables

High-risk fertility behaviour (yes, no) was considered the primary exposure variable. The variable was created based on three high-risk fertility markers (with four indicators) as per the relevant international guidelines: (i) age at pregnancy, (ii) interval between indexed pregnancy and the most earlier pregnancy, and (iii) total number of children ever born. A positive response on high-risk fertility behaviour was considered if the pregnancy occurred before age 18 years or after age 34 years, there was less than a 24 month interval between two subsequent pregnancies or if women had at least two children before the indexed child. We also generated a number of sub-variables based on the number of risky behaviors reported by the women. These were: (i) number of high-risk fertility behaviours (no, single, multiple) and no high-risk fertility behaviour (yes, no).

Covariates

We considered a range of individual-, household-, and community-level factors as potentially influencing the relationship between high-risk fertility behaviour and perinatal mortality. The covariates were selected in two stages. We first generated a list of all potential covariates by

reviewing the relevant literature for LMICs, in particular Bangladesh and its neighbouring countries [4,14,18-24]. The availability of the selected potential variables was then checked in the survey we analysed. The variables available in the survey, as well as the exposure variable considered, were then checked for multicollinearity and highly correlated variables were deleted. Variables that met these criteria were finally selected to be included in this study and categorized as individual-, household-, and community-level factors. Individual-level factors were mothers' educational status (no education, primary, secondary and higher) and mothers' working status (yes, no). Household-level factors were mothers' partners' education (no education, primary, secondary and higher), partner's occupation (agricultural worker, blue collar worker [rickshaw driver, brick field worker, domestic servant, non-agricultural worker, carpenter, mason etc.), white collar worker [doctor, lawyer, dentists], pink collar worker [big businessmen, small business/trader] and others [not working, unemployed, retired etc.) and wealth quintile (poorest, poorer, middle, richer and richest). Place of residence (urban, rural) and region of the place of residence (Barishal, Chattogram, Dhaka, Khulna, Mymensingh, Rajshahi, Rangpur, and Sylhet) were included as community-level factors. The wealth quintile variable was created by the BDHS using principal component analysis (PCA), which incorporated variables related to household assets, such as roof type or ownership of a radio/television. The PCA generated a score that was divided into five equal parts, with the highest score representing the richest quintile and the lowest score representing the poorest quintile.

Statistical analysis

Descriptive statistics were used to characterise the respondents. Mixed-effect multilevel logistic regression models were then used to explore the association between the exposure and outcome variables adjusting for individual-, household-, and community-level factors. Mixed effect multilevel logistic regression model was chosen due to the hierarchical structure of the BDHS data, in which individuals are nested within a household and households are nested within a cluster. Survey data with such a structure creates multiple levels of dependencies, such as respondents from the same household behave similarly and respondents from the same PSU behave similarly.

This creates multiple levels of dependencies in the survey data [26]. Both unadjusted and adjusted models were run. In the unadjusted model, a particular exposure variable was considered with the outcome variable whereas in the adjusted model exposure and outcome variable was considered with all covariates. We employed a progressive model building technique and ran a total of three models. Model 1 consisted solely of individual-level factors, while Model 2 incorporated both individual and household-level factors. Finally, Model 3, the final iteration, included individual, household, and community-level factors. The complex survey design was considered in all analyses. Results are reported as Odds Ratios (OR) with 95% Confidence Intervals (95% CI). Statistical package R (version 4.1) was used for all statistical analyses.

Ethics approval and consent to participate: The survey analysed was approved by the institutional review board of ICF and the National Research Ethics Committee of the Bangladesh Medical Research Council. Informed consent was obtained from all participants. All necessary patient/participant consent has been obtained and the appropriate institutional forms have been archived. No separate ethical approval was required to conduct this study. We obtained permission to access this survey and conduct this research. All methods were performed in accordance with the relevant guidelines and regulations.

Results

Participant characteristics

The distribution of the respondents analysed in this study as well as perinatal mortality across individual-, household-, and community-level characteristics is shown in Table 1. The majority of respondents analysed were aged between 20 and 34 years (85.8%) at the indexed pregnancy and possessed secondary level education (48.3%). Around 52% of the total respondents' husbands were blue collar workers, followed by pink (21%) and agricultural workers (20%). More than two-thirds of the respondents were from rural areas and around one-quarter of respondents were from the Dhaka region.

Distribution of perinatal mortality across individual-, household-, and community-level characteristics

The average rate of perinatal mortality was 48.0 per 1000 live bi[illegible] (Table 1). A higher prevalence of perinatal mortality (132.8 per 1000 live births) was found among mothers aged >34 years following 52.8 per 1,000 live birth among mothers aged <18 years. Prevalence of perinatal mortality was found higher among mothers with primary education (59 per 1,000 live births) and those engaged in formal income-generating activities (54 per 1,000 live births).

Table 1: Sample characteristics of number of pregnancies of 7+ months duration and distribution of perinatal mortality (per 1000 live births) in Bangladesh

**Characteristics**	**Prevalence, 95% CI**	**Perinatal mortality per 1000 live births (95% CI)**
**Overall**		**48.0 (43.0-53.4)**
**Mothers age at the indexed birth**		
<18	9.4 (8.7-10.2)	52.4 (38.1-71.7)
20-34	85.8 (84.9-86.6)	42.7 (37.9-48.2)
≥35	4.8 (4.3-5.3)	132.8 (98.5-176.6)
**Mother's educational status**		
No education	7.3 (6.5-8.3)	40.1 (25.7-62.1)
Primary	29.1 (27.4-30.7)	58.6 (48.7-70.3)
Secondary	48.3 (46.7-49.9)	45.3 (38.7-53.0)
Higher	15.3 (14.1-16.6)	39.9 (29.7-53.4)
**Mother's working status**		
Yes	40.8 (38.7-42.9)	53.7 (45.6-63.3)
No	59.2 (57.1-61.3)	44.0 (38.3-50.5)
**Partner's occupation**		
Agricultural worker	20.3 (18.8-21.9)	43.6 (34.8-54.6)
Blue color worker	52.1 (50.4-53.8)	53.5 (46.5-61.5)
White color worker	5.6 (4.9-6.3)	34.8 (19.7-60.9)
Pink color worker	21.1 (19.9-22.4)	41.4 (32.4-52.7)
Others	0.94 (0.7-1.24)	50.9 (16.5-146.2)
**Wealth quintile**		
Poorest	21.5 (19.4-23.7)	51.9 (41.9-64.1)
Poorer	20.4 (19.0-21.8)	49.6 (39.7-61.7)
Middle	18.8 (17.5-20.2)	50.3 (39.4-64.1)
Richer	20.1 (18.6-21.8)	48.9 (38.5-61.9)
Richest	19.2 (17.5-20.9)	38.5 (29.2-50.7)

**Place of residence**		
Urban	27.4 (25.9-28.9)	48.6 (39.9-58.9)
Rural	72.6 (71.1-74.1)	47.7 (41.9-54.4)
**Region of the place of residence**		
Barishal	5.5 (5.1-6.1)	45.3 (33.3-61.3)
Chattogram	20.9 (19.5-22.5)	52.8 (41.6-66.7)
Dhaka	25.9 (24.3-27.5)	47.2 (37.0-60.0)
Khulna	9.1 (8.4-9.9)	40.5 (26.2-62.0)
Mymensingh	8.4 (7.7-9.2)	51.9 (40.5-66.3)
Rajshahi	11.7 (10.6-12.9)	53.3 (39.7-71.2)
Rangpur	10.3 (9.5-11.2)	44.8 (30.8-64.9)
Sylhet	8.1 (7.3-9.1)	40.6 (30.8-53.3)

Around 54 per 1,000 perinatal mortality was reported among women whose partners were blue collar workers. Prevalence of perinatal mortality was found to be higher among mothers in the poorest wealth quintile (52 per 1,000 live birth) followed by the poorer wealth quintile (50 per 1,000 live births) and the middle wealth quintile (51 per 1,000 live births). A similar prevalence of perinatal mortality was reported between mothers from rural and urban areas although Chattogram and Rajshahi regions had the highest prevalence of perinatal mortality with around 53 deaths per 1,000 live births.

High-risk fertility behaviour

Of the women's data analysed, around 46% of the births in the five years preceding the survey were in any-high risk category (Table 2). Of them, a single high-risk fertility behaviour was reported by 38% of total respondents and multiple high-risk fertility behaviour was reported by 8%. The most common form of single high-risk fertility behaviour was birth order above 2 (>2 children ever born, 61%) following pregnancy aged less than 18 years (23%) and a birth interval of less than 24 months (14%). The most common form of multiple high-risk fertility behaviour was pregnancy aged above 34 years and birth order above 2 (48%) followed by birth interval less than 24 months and birth order above 2 (42%). Prevalence of perinatal mortality was found to be higher among women with any high risk fertility behaviour (8.5% *vs* 1.7%). The prevalence was found to be even

higher among women with multiple high risk fertility behaviours (12.8%) than single high risk fertility behaviour (7.5%).

Table 2: Pattern of higher risk fertility behaviour and distribution of perinatal mortality across pattern of higher risk fertility behaviour in Bangladesh

	**High risk fertility behaviour**		**Perinatal mortality**	
	Weighted frequency	Prevalence, 95% CI	Weighted frequency	Prevalence, 95% CI
**High risk fertility behaviour**				
No	4816	53.9 (52.5-55.4)	80	1.7 (1.3-2.1)
Yes	4115	46.1 (44.7-47.5)	349	8.5 (7.5-9.5)
**Single high risk fertility behaviour**	**3378**	**37.8 (36.5-39.2)**	**254**	**7.5 (6.6-8.6)**
Age less than 18 years	765	22.7 (21.03-24.4)	11	1.38 (0.06-3.0)
Age above 34 years	56	1.7 (1.3-2.2)	2	3.6 (0.07-14.7)
The birth interval of less than 24 months	479	14.2 (12.5-16.0)	55	11.4 (8.5-15.1)
Birth order above 2	2078	60.8 (58.6-63.0)	165	8.0 (6.8-9.5)
**Multiple high risk fertility behaviour**	**737**	**8.3 (7.5-9.1)**	**94**	**12.8 (10.0-16.2)**
Age less than 18 years and birth interval less than 24 months	54	7.3 (4.9-10.8)	12	21.6 (12.6-34.7)
Age above 34 years and birth interval less than 24 months	4	0.1 (0.01-1.89)	1	4.4 (0.04-32.0)
Age above 34 years and birth order above 2	354	48.0 (43.4-52.7)	55	15.4 (11.4-20.6)
Age above 34 years and birth interval less than 24 months, and birth order above 2	14	2.0 (0.09-4.2)	0	0
Birth interval less than 24 months and birth order above 2	311	42.2 (37.5-47.0)	28	8.9 (5.4-14.3)
**No high-risk fertility behaviour**	2402	26.9 (25.9-27.9)	53	2.2 (1.6-3.0)

Relationship between high-risk fertility and perinatal mortality

The results of the mixed-effect multilevel logistic regression model to explore the association of perinatal mortality with high-risk fertility behaviour, the number of high-risk fertility behaviour, and no high-risk fertility behaviour are presented in Table 3 and supplementary tables 1-3. The likelihood of perinatal mortality was found to be 1.87 times higher (aOR, 1.87, 95% CI, 1.61-2.14) among mothers with any high-risk fertility behaviours as compared to the mothers without any high-risk fertility behaviour. Compared to the women with no high risk fertility behavior, the likelihood of perinatal mortality was found to be higher among women with single high-risk fertility behaviour (aOR, 1.77, 95% CI, 1.50-2.05) and multiple high-risk fertility behaviours (aOR, 2.30, 95% CI, 1.96-2.64). Additionally, likelihood of perinatal mortality was found 64% (aOR, 0.27-0.49) lower among mothers having no high-risk fertility behaviour as compared to the mothers having any high-risk fertility behaviour.

Table 3: Unadjusted and adjusted associations of perinatal mortality with high-risk fertility behaviour, number of high-risk fertility behaviour, unavoidable high-risk fertility behaviour, and no high-risk fertility behaviour, Bangladesh 2017/18

Characteristics	**Unadjusted association**		**Adjusted association**++	
	OR (95% CI)	*p-value*	OR (95% CI)	*p-value*
**High risk fertility behaviour**				
No (ref)	1.00		1.00	
Yes	5.83 (4.52-7.52)	0.000	1.87 (1.61-2.14)	0.000
**Number of high-risk fertility behaviour**				
No (ref)	1.00		1.00	
Single	5.37 (4.13-6.97)	0.000	1.77 (1.50-2.05)	0.000
Multiple	8.00 (5.78-11.09)	0.000	2.30 (1.96-2.64)	0.000
**No high-risk fertility behavior**				
No (ref)	1.00		1.00	
Yes	0.39 (0.29-0.52)	0.000	0.36 (0.27-0.49)	0.000

Notes: Models are adjusted with respondents' education, respondents' works, wealth quintile, respondents' husband occupation, place of residence and region of place of residence

Discussion

In our analysis of 2017/18 BDHS data, we found that around 46% of the total births that occurred within five years preceding the survey were in any-high risk category, including 38% and 8% of the total births occurred with single and multiple high-risk category, respectively. The results demonstrate that women's high-risk fertility behaviour increases the occurrence of perinatal mortality in general and is further compounded with increasing numbers of high-risk fertility behaviours. This indicates that attaining the SDG targets related to neonatal and under-five mortality are improbable for Bangladesh where around half of the total births occur among women whose pregnancies involve single or multiple high-risk fertility behaviours. These findings highlight the need for national-level policies and programs to reduce pregnancy with high-risk fertility behaviours of mothers and continuity of maternal healthcare services use.

The prevalence of high-risk fertility behaviour found in this study for Bangladesh is higher than the other south Asian countries [18,27], but lower than in African countries [28]. However, an important observation is that the prevalence of high-risk fertility behaviour reported in this study is higher than the previously reported prevalence for Bangladesh (36%) [10]. A similar rising pattern has been observed in other LMICs. This is concerning considering significant public health measures have been taken in LMICs to improve maternal and child health, particularly the MDGs period which ended only three years prior to the time of this survey [29]. This rising prevalence could be linked with the recent patterning of fertility in LMICs such as Bangladesh. For instance, total fertility rate was high in Bangladesh a decade ago, as such there remains a higher percentage of reproductive-aged women [30]. Among these women, early marriage and early child-bearing are still highly prevalent and due to such early initation into reproductive life, these women have a longer period of child-bearing [31]. This presents issues such as higher parity and close birth spacing. Despite this, pregnancy at later ages is also increasing rapidly in LMICs such as Bangladesh due to delayed starting of conjugal life, mostly because of the increasing age at marriage, women's empowerment,

and higher prevalence of unmet need for family planning and contracrption [32]. These issues are directly contributing to the rising prevalence of high-risk fertility in Bangladesh.

In our analysis, the odds of perinatal mortality was found to be around 2 times higher among women with any high-risk fertility behaviours after controlling for potential confounders and this was found to increase further with the number of high-risk fertility behaviours. These observations are particularly novel for LMICs. As such, we are unable not make direct comparisons. However, available studies in LMICs and Bangladesh have found individual high-risk fertility to be linked with several adverse child health outcomes, including under-five mortality [14,21]. Importantly, each type of high-risk fertility behaviour has been linked to numerous adverse characteristics which later lead to the occurrence of perinatal mortality.

For instance, women who become pregnant at an early age may not have matured physically to allow for optimal foetal development [21,33] and they may not aware of their upcoming child health issues [34]. This also increases the occurrence of unintentional injury [35]. Moreover, low maternal healthcare service use and in particular continuity of care has been found to be higher among younger and older aged mothers [36]. This has been attributed to factors such as inadequate knowledge about pregnancy, the importance of maternal healthcare services use, lower empowerment, lower autonomy to access maternal healthcare services and tendency to depend on prior pregnancy experiences [36,37]. Because of these charecteristics, as well as lower and higher age of women, are also found to be associated with experiencing unintended pregnancy, which accounts for a quarter of live births in Bangladesh [31]. Unintended pregnancy also contributes numerous adverse consequences and lower use of maternal healthcare services [31]. Also, pregnancy among women later in their reproductive lives are at increased risk for congenital and chromosomal abnormalities [21,33]. Short interval birth could also be linked with perinatal mortality in several ways. For instance, mothers with a short birth interval cannot recover their nutritional stores which may result in malnutrition in the next pregnancy [38]. It also increases the risks of

placenta abruption and premature rupture of the membrane [39]. Higher parity, among which the occurrence of birth in short interval, is associated with maternal undernutrition and lower access or maternal healthcare services [40]. These characteristics thereby increase the risk of adverse pregnancy outcomes, including anaemia, intrauterine growth retardation, preterm birth, and low birth weight [14,21,34,41]. Importantly, the extent of these adverse outcomes increase with the increasing number of high-risk ferility behaviours and for particular risk factors such as early or late aged pregnancy. Such lower use of healthcare services use along with continuity, higher occurrence of unwanted pregnancy as well as other adverse charecteristics later contribute to increase occurence of perinatal mortality.

To address the burden of high-risk fertility behaviour and related adverse consequences in Bangladesh, prevention, protection and treatment strategies need to be focused in the relevant policies and programs which are currently absent. For this to happen, mothers in any of the high-risk categories should be counselled appropriately about their potential risk of getting pregnancy and having baby if they became pregnant and have a baby. Improving the uptake of maternal and postnatal healthcare services are also key to the prevention of neonatal and infant mortality. Access to family planning services and use of contraception, including access to contraception at the post-partum period, should also be ensured, particularly among the disadvantaged and unaware population where occurrence of birth in short interval is very higher [16,42]. Existing health problems, including maternal undernutrition and anaemia, are required to be addressed properly. For this, screening programs need to be ensured before pregnancy through community level family planning workers as well as during pregnancy when women accessing preconception care and treat them. Finally, maternal healthcare services, as well as the continuity of using maternal healthcare services, should be ensured for all women, particularly, for women having any high-risk fertility behaviour.

The findings of this study should be interpreted while taking into account its limitations. First, the findings of this study were generated based on analysing a cross-sectional survey, therefore, they are correlational only rather than casual. Second, data recorded in the survey analysed were collected based on the mothers' retrospective responses to the events that occurred up to five years back. This increased the risk of recall bias, although during data collection the respondents were asked multiple questions which helped the survey to reduce the recall bias. Third, this study does not incorporate healthcare facility-level factors in the models, though a broad range of individual, household and community level covariates were adjusted. Since healthcare facilities place an important role in the occurrence of perinatal mortality, such a lack could deter the strengths of the reported association between exposure and outcome variable. Similarly, mothers' physical and behavioural characteristics at the time of indexed pregnancy and maternal healthcare services use as well as continuity of using maternal healthcare services are important factors to consider in the model to determine a precise estimate of the reported association. Also, this study focused exclusively high-risk behaviours among women. As women's partners play a key role in family planning (or lack thereof), understanding the role partner attitudes regarding fertility and family planning are needed to in future studies. Despite this, the findings of this study are robust as they were generated by analysing nationally representative survey data with a quite large sample size. Advanced statistical modelling technique was used to determine the targeted associations. Therefore, the findings of this study will be helpful for evidence-based policy and program making. This would ultimately help LIMCs and Bangladesh in reducing the prevalence of high-risk fertility behaviour and perinatal mortality which are imperative to achieving the relevant SDGs targets by 2030.

Conclusions

High-risk fertility behaviour was reported by almost half of mothers in this study with, 38% reporting single high-risk fertility behaviour and 8% reporting multiple high-risk fertility behaviour. We found a high odds of perinatal mortality among mothers having any high-risk fertility

behaviours and the odds were found to be increased with the increased number of high-risk fertility behaviour of mothers. Such a higher prevalence of high-risk fertility behaviour along with increased perinatal mortality among this population indicate it is improbable for Bangladesh to achieve SDG targets of reducing neonatal and under-five mortality to 12 and 25 per 1000 live births by 2030. Maternal healthcare services use with continuity are important to reduce occurrence of high-risk ferility and related adverse outcomes including perinatal mortality. Policy and program development is also needed reduce the prevalence of high-risk fertility behaviour through education about the adverse effects of high-risk fertility behaviour as well as ensuring proper family planning services and contraception in preconception and post-partum period. These should be an important pathway to achieving the SDGs goals by 2030 and ensuring positive outcomes for both women and children.

Declaration of interests: The authors declare that they have no known competing financial interests or personal relationships that could have appeared to influence the work reported in this paper.

Acknowledgements: We acknowledge the DHS of the USA for approving us to use their data. Melissa L Harris is supported by a Glady M. Brawn Fellowship.

Funding: The authors received no specific funding for this work.

Authors' contributions: Khan MN designed the study, performed the data analysis, and wrote the first draft of this manuscript. Harris ML critically reviewed and edited all versions of this manuscript. All authors approved this final version of the manuscript.

Competing of interest: None.

Consent for publication: Informed consent was obtained from all participants during the survey using standard DHS consent form.

Data availability: The datasets used and analyzed in this study are available from the Measure DHS website: https://dhsprogram.com/data/available-datasets.cfm

References

1 Organization., W. H. (World Health Organization. , Geneva, Switzarland 2021).

2 Organization., W. H. (World Health Organization. , Geneva, Switzarland 2021).

3 Hug, L., Alexander, M., You, D., Alkema, L. & for Child, U. I.-a. G. National, regional, and global levels and trends in neonatal mortality between 1990 and 2017, with scenario-based projections to 2030: a systematic analysis. *The Lancet Global Health* **7**, e710-e720 (2019).

4 Hossain, M. B., Kanti Mistry, S., Mohsin, M. & Rahaman Khan, M. H. Trends and determinants of perinatal mortality in Bangladesh. *PloS one* **14**, e0221503 (2019).

5 UNICEF. (UNICEF, , 2020).

6 Lassi, Z. S. & Bhutta, Z. A. Community-based intervention packages for reducing maternal and neonatal morbidity and mortality and improving neonatal outcomes. *Cochrane database of systematic reviews* (2015).

7 Bhutta, Z. A. *et al.* Can available interventions end preventable deaths in mothers, newborn babies, and stillbirths, and at what cost? *The Lancet* **384**, 347-370 (2014).

8 Dodd, R. & Cassels, A. Health, development and the millennium development goals. *Annals of Tropical Medicine & Parasitology* **100**, 379-387 (2006).

9 Kekki, P. Primary health care and the Millennium Development Goals: issues for discussion. *Geneva: WHO* (2004).

10 Khan, M. A. *et al.* Trends and projections of under-5 mortality in Bangladesh including the effects of maternal high-risk fertility behaviours and use of healthcare services. *PloS one* **16**, e0246210 (2021).

11 Roro, E. M., Sisay, M. M. & Sibley, L. M. Determinants of perinatal mortality among cohorts of pregnant women in three districts of North Showa zone, Oromia Region, Ethiopia: Community based nested case control study. *BMC public health* **18**, 1-11 (2018).

12 Heen, E., Størdal, K., Wachira, J., Heen, I. & Lundeby, K. M. Do we need a new global policy for ending preventable perinatal deaths in fragile low-income countries? *Journal of Global Health* **12** (2022).

13 Khan, M. N., Trisha, N. I. & Rashid, M. M. Availability and readiness of healthcare facilities and their effects on under-five mortality in Bangladesh: Analysis of linked data. *Journal of global health* **2022** (2022).

14 Amir-ud-Din, R., Naz, L., Rubi, A., Usman, M. & Ghimire, U. Impact of high-risk fertility behaviours on underfive mortality in Asia and Africa: evidence from Demographic and Health Surveys. *BMC pregnancy and childbirth* **21**, 1-14 (2021).

15 UNICEF. Ending child marriage: A profile of progress in Bangladesh (United Nations Children's Fund 2020).

16 Islam, M. Z., Islam, M. M., Rahman, M. & Khan, M. Exploring hot spots of short birth intervals and associated factors using a nationally representative survey in Bangladesh. *Scientific Reports* **12**, 1-10 (2022).

17 Abedin, S. & Arunachalam, D. Maternal autonomy and high-risk pregnancy in Bangladesh: the mediating influences of childbearing practices and antenatal care. *BMC Pregnancy and Childbirth* **20**, 1-8 (2020).

18 Pal, S. K. & Shekhar, C. Examining the role of high-risk fertility behaviour in chronic undernutrition among Indian married women age 15-49. *Clinical Epidemiology and Global Health* **11**, 100739 (2021).

19 Rahman, M. *et al.* Association between high-risk fertility behaviours and the likelihood of chronic undernutrition and anaemia among married Bangladeshi women of reproductive age. *Public health nutrition* **20**, 305-314 (2017).

20 Rahman, M., Hosen, A. & Khan, M. A. Association between maternal high-risk fertility behavior and childhood morbidity in Bangladesh: a nationally representative cross-sectional survey. *The American journal of tropical medicine and hygiene* **101**, 929 (2019).

21 Asresie, M. B. & Dagnew, G. W. Association of maternal high-risk fertility behavior and under-five mortality in Ethiopia: Community-based survey. *Plos one* **17**, e0267802 (2022).

22 Fawole, A. O. *et al.* Determinants of perinatal mortality in Nigeria. *International Journal of Gynecology & Obstetrics* **114**, 37-42 (2011).

23 Getiye, Y. & Fantahun, M. Factors associated with perinatal mortality among public health deliveries in Addis Ababa, Ethiopia, an unmatched case control study. *BMC pregnancy and childbirth* **17**, 1-7 (2017).

24 Debelew, G. T. Magnitude and determinants of perinatal mortality in Southwest Ethiopia. *Journal of Pregnancy* **2020** (2020).

25 Murray, S., MacKay, D., Stock, S., Pell, J. & Norman, J. Association of gestational age at birth with risk of perinatal mortality and special educational need among twins. *JAMA pediatrics* **174**, 437-445 (2020).

26 Diez-Roux, A. V. Multilevel analysis in public health research. *Annual review of public health* **21**, 171-192 (2000).

27 Rahman, M. *et al.* Maternal high-risk fertility behavior and association with chronic undernutrition among children under age 5 y in India, Bangladesh, and Nepal: do poor children have a higher risk? *Nutrition* **49**, 32-40 (2018).

28 Tamirat, K. S., Tesema, G. A. & Tessema, Z. T. Determinants of maternal high-risk fertility behaviors and its correlation with child stunting and anemia in the East Africa region: A pooled analysis of nine East African countries. *PloS one* **16**, e0253736 (2021).

29 Beattie, R., Brown, N. & Cass, H. Millennium development goals progress report. *Archives of disease in childhood* **100**, S1-S1 (2015).

30 National Institute of Population Research and Training (NIPORT) and ICF. Bangladesh Demography and Health Survey, 2017/18. (NIPORT, ACPR, and ICF., 2020).

31 KHAN, M. N. Effects of Unintended Pregnancy on Maternal Healthcare Services Use in Bangladesh. *Faculty of Health and Medicine, School of Medicine and Public Health, The* **1** (2020).

32 Haq, I., Alam, M. & Methun, I. H. Contributions of proximate determinants to fertility transition in Bangladesh: an analysis of Bongaarts' fertility model. *International Journal of Travel Medicine and Global Health* **7**, 23-32 (2019).

33 de Vienne, C. M., Creveuil, C. & Dreyfus, M. Does young maternal age increase the risk of adverse obstetric, fetal and neonatal outcomes: a cohort study. *European Journal of Obstetrics & Gynecology and Reproductive Biology* **147**, 151-156 (2009).

34 Chen, X.-K. *et al.* Teenage pregnancy and adverse birth outcomes: a large population based retrospective cohort study. *International journal of epidemiology* **36**, 368-373 (2007).

35 Yao, M., Wu, G., Zhao, Z., Luo, M. & Zhang, J. Unintentional injury mortality among children under age five in urban and rural areas in the Sichuan province of west China, 2009–2017. *Scientific reports* **9**, 1-8 (2019).

36 Khan, M. N., Harris, M. L. & Loxton, D. Assessing the effect of pregnancy intention at conception on the continuum of care in maternal healthcare services use in Bangladesh: Evidence from a nationally representative cross-sectional survey. *PLoS ONE* **15**, e0242729 (2020).

37 Andriani, H., Rachmadani, S. D., Natasha, V. & Saptari, A. Continuity of maternal healthcare services utilisation in Indonesia: analysis of determinants from the Indonesia Demographic and Health Survey. *Family Medicine and Community Health* **9** (2021).

38 Chungkham, H. S., Sahoo, H. & Marbaniang, S. P. Birth interval and childhood undernutrition: Evidence from a large scale survey in India. *Clinical Epidemiology and Global Health* **8**, 1189-1194 (2020).

39 Jena, B. H., Biks, G. A., Gete, Y. K. & Gelaye, K. A. Incidence of preterm premature rupture of membranes and its association with inter-pregnancy interval: a prospective cohort study. *Scientific reports* **12**, 1-8 (2022).

40 Kumera, G., Gedle, D., Alebel, A., Feyera, F. & Eshetie, S. Undernutrition and its association with socio-demographic, anemia and intestinal parasitic infection among pregnant women attending antenatal care at the University of Gondar Hospital, Northwest Ethiopia. *Maternal health, neonatology and perinatology* **4**, 1-10 (2018).

41 Ganchimeg, T. *et al.* Pregnancy and childbirth outcomes among adolescent mothers: a W orld H ealth O rganization multicountry study. *BJOG: An International Journal of Obstetrics & Gynaecology* **121**, 40-48 (2014).

42 Islam, M. Z., Islam, M. M., Rahman, M. M. & Khan, M. N. Prevalence and risk factors of short birth interval in Bangladesh: Evidence from the linked data of population and health facility survey. *PLOS Global Public Health* **2**, e0000288 (2022).

Supplementary tables

Click here to access/download
Supporting Information
Supplementary table.docx
