# Supplementary material for: Association between maternal high-risk fertility behaviour and perinatal mortality in Bangladesh: Evidence from the Demographic and Health Survey
Source: PLoS One. 2023 Nov 27;18(11):e0294464. doi: 10.1371/journal.pone.0294464 (PMC10681254; doi:10.1371/journal.pone.0294464)
Supplement: Attachment — Submitted filename: Response to reviewers.docx [file pone.0294464.s005.docx]

**Reviewers’ comments and authors’ responses**

| **Reviewers’ comments** | **Authors’ response** | **Page** |
| --- | --- | --- |
| **Reviewer#1** |  |  |
| The article is very well-written, provides background information in a good manner, methods and results are explained well and discussed accordingly. I have several points to highlight: | Thank you for your positive comments regarding our paper We have addressed each of your comments and provide a point-by-point response below. |  |
| 1. It is always important to know what are the basis of choosing the confounders, especially when it comes for the outcome as PM, I would prefer to have a paragraph in the methods explaining the reasons of choosing one or another confounder for adjusting. I also think age is an important factor to take into account, rather than working status, which is affected by age. | We have a separate section in the methods section named “covariates” where we discuss broadly the covariate selection procedure. Specifically, we have the following sentences-  *The covariates were selected in two stages. We first generated a list of all potential covariates by reviewing the relevant literature for LMICs, in particular Bangladesh, and its neighbouring countries ^4,14,18-24^. The availability of the selected potential variables was then checked against the variables available for analysis. These variables, as well as the exposure variables, were checked for multicollinearity and highly correlated variables were deleted. Variables that met these criteria were then categorized as individual-, household-, and community-level factors. Individual-level factors were…………*  We concur with the reviewer's perspective on the significance of considering age, especially when examining PM as the primary outcome variable. However, it's worth noting that our exposure variable already encompasses an age component, given that the age of pregnancy is a critical factor in defining high-risk fertility. Consequently, if we were to reintroduce age as a variable in the model, it could lead to very high multicollinearity. This is why we excluded the variable “age” from the analysis. |  |
| 2. The PM rate needs to be counted per 1000 births and not live births, its important and please have a close look at the manuscript for each place authors are referring PM rate. | Thank you for bringing this to our attention. We have calculated the rate for all births (per 1,000). We acknowledge that there was an error in our presentation where we incorrectly stated live births only. We have taken steps to rectify this issue by editing the relevant sentences. |  |
| 3. In my mind, there are lack of discussion on characteristics, while there are many things presented in characteristics table in results, it would be better for article quality if authors try and explain some of the differences in PM rates for different characteristics, like region. | Thank you for your observation. We have now revised the relevant sections of the discussion section and discussed several characteristics, including rurality, region of residence, healthcare services access and availability. |  |
| Overall, I think the research is well-conducted and needs to be published. | Many thanks. |  |
| **Reviewer#2** |  |  |
| This is a manuscript with a relevant theme for public health, with a clear and objective presentation, current theoretical framework. It presents a well-contextualized introduction section and a clear objective. In the method section, it brings the necessary details for understanding the research scenario, data collection and study variables, as well as presenting the data analysis with consistency between the chosen tests. | Many thanks for your positive response. |  |
| In the result section, clear texts and with notes without repetitions of everything that is in the tables. The tables are well organized, but I suggest just including the year and place in the description. | We have added year and place of data collection in the table titles as requested. |  |
| As for the discussion section, the authors bring counterpoints in relation to the evidenced results, leaving a series of reflections to the reader about the findings; important reflections so that one can think of strategies for discussions about public policies and decision-making. | No specific change is required to address this point. |  |
| In conclusion, it responds to the proposed objective and brings important inferences that reveal the relevance of the study. | No specific change is required to address this point. |  |
